# A case of marked alveolar bone augmentation after chemotherapy in a pediatric patient with acute lymphocytic leukemia

**DOI:** 10.1186/s12903-026-07998-0

**Published:** 2026-03-26

**Authors:** Shinya Koshinuma, Yasuyuki Asada, Takafumi Fujii, Rina Hirai, Takato Murai, Kazuki Takaoka

**Affiliations:** 1 Department of Oral and Maxillofacial Surgery, Shiga University of Medical Science, Ōtsu, 520-2192 Japan; 2 Department of Oral and Maxillofacial Surgery, Nagahama Japan Red Cross Hospital, Nagahama city, Japan; 3 Department of Oral and Maxillofacial Surgery, Toyosato Hospital, Inukami, Japan

**Keywords:** Bone resorption, Alveolar bone, Acute lymphoblastic leukemia, Pediatric cancer

## Abstract

**Background:**

Leukemia is correlated with alveolar bone loss, while remission is reportedly associated with an increase in alveolar bone volume. However, there have been no reports from Japan detailing substantial recovery of the alveolar bone following remission from leukemia.

**Case presentation:**

A 14-year-old male patient presented to our clinic with fatigue, a low-grade fever, and bilateral mandibular molar tooth movement with gingival swelling around the molars that affected his ability to eat. Panoramic radiography revealed alveolar bone resorption in both jaws, while the alveolar hard line, mandibular canal wall, and trabecular structure were indistinct. The patient had an elevated white blood cell count and blasts were observed in the peripheral blood. Additionally, C-reactive protein, lactate dehydrogenase, and ferritin levels were elevated. These findings suggested leukemia. Bone marrow biopsy confirmed acute lymphocytic leukemia (ALL). ALL invasion had caused alveolar bone resorption and loose teeth, resulting in difficulty eating. He was started on IA4 remission induction therapy and consolidation therapy. The patient experienced successful remission.

**Conclusions:**

After chemotherapy, the patient recovered oral function, which allowed him to resume normal food intake. Notably, the alveolar bone recovered considerably and tooth movement disappeared. Clinicians should consider alveolar bone resorption and tooth mobility as potential early signs of ALL in pediatric patients and recognize that timely chemotherapy can offer substantial oral recovery and alveolar bone regeneration.

## Key Message

We report a case of a pediatric patient with alveolar bone resorption due to acute lymphocytic leukemia who developed difficulty eating. After chemotherapy, the alveolar bone regenerated, and the patient regained his ability to eat.

## Background

Leukemia accounts for 38% of childhood cancers in Japan. Among acute lymphoblastic leukemia (ALL) subtypes, B-cell (B-ALL) and T-cell acute lymphoblastic leukemia account for 80–85% and 10–15% of cases, respectively [1].

Approximately 20–35% of patients with leukemia initially have oral symptoms, particularly gingival hemorrhage or swelling, followed by jawbone abnormalities such as miconus hypersensitivity, alveolar bone resorption, and tooth movement; therefore, many leukemia cases have been detected by oral signs [2–8].

Radiographic studies show ALL is correlated with mild or worsening jaw bone symptoms in pediatric patients [9]. However, no reports of jaw bone resorption in patients with ALL in Japan exist, although jaw bone resorption has been observed due to leukemia cell infiltration [10].

Here, we report a pediatric case of ALL featuring significant jaw bone resorption due to leukemia cell infiltration, resulting in severe tooth movement and loss of proper jaw occlusion. We detail the patient’s recovery following remission.

## Case presentation

A 14-year-old male patient presented to our clinic on Oct 12th, 2021, with gingival hemorrhage and full maxillary tooth movement, leading to feeding difficulties. His family and medical histories were unremarkable. In late September 2021, he had noticed bilateral mandibular molar tooth movement and gingival swelling. Despite occlusal adjustment by a local dentist, symptoms did not improve, gingival swelling spread to the entire jaw, and he began to bleed easily.

Upon examination, the patient had a body temperature of 37.4 °C, was feeling fatigued, and was severely underweight (height, 162 cm; weight, 38.5 kg; body mass index, 14.7 kg/m^2^). He reported that he could only take soft food orally.

**Fig. 1:**
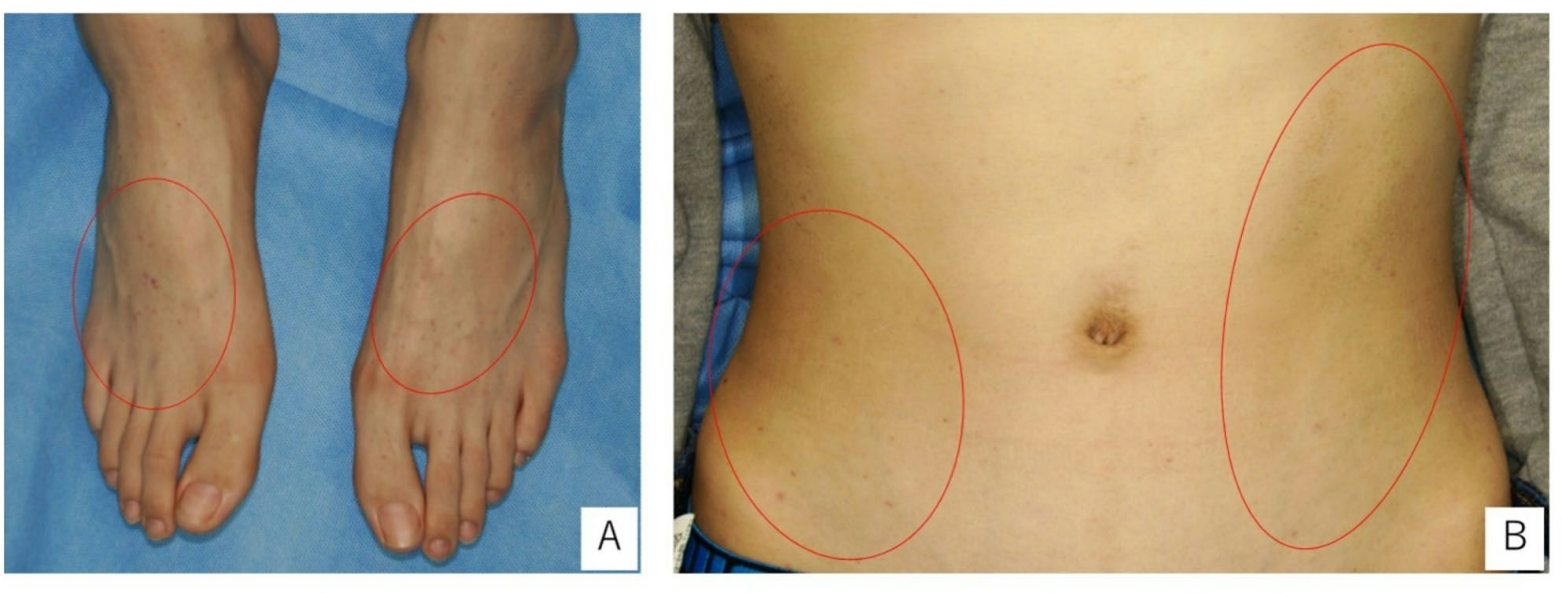
Dorsal foot, abdominal portrait. **A** Photograph of the dorsal foot. **B** Photograph of the abdomen. Scattered petechial hemorrhagic patches were observed on the dorsal surface of the foot and abdomen

He had pain bilaterally in the knees, elbows, and shoulder joints. Scattered petechial hemorrhagic patches were observed on the dorsal surfaces of his feet and abdomen (Fig. 1), and his liver and spleen were enlarged on palpation.

**Fig. 2:**
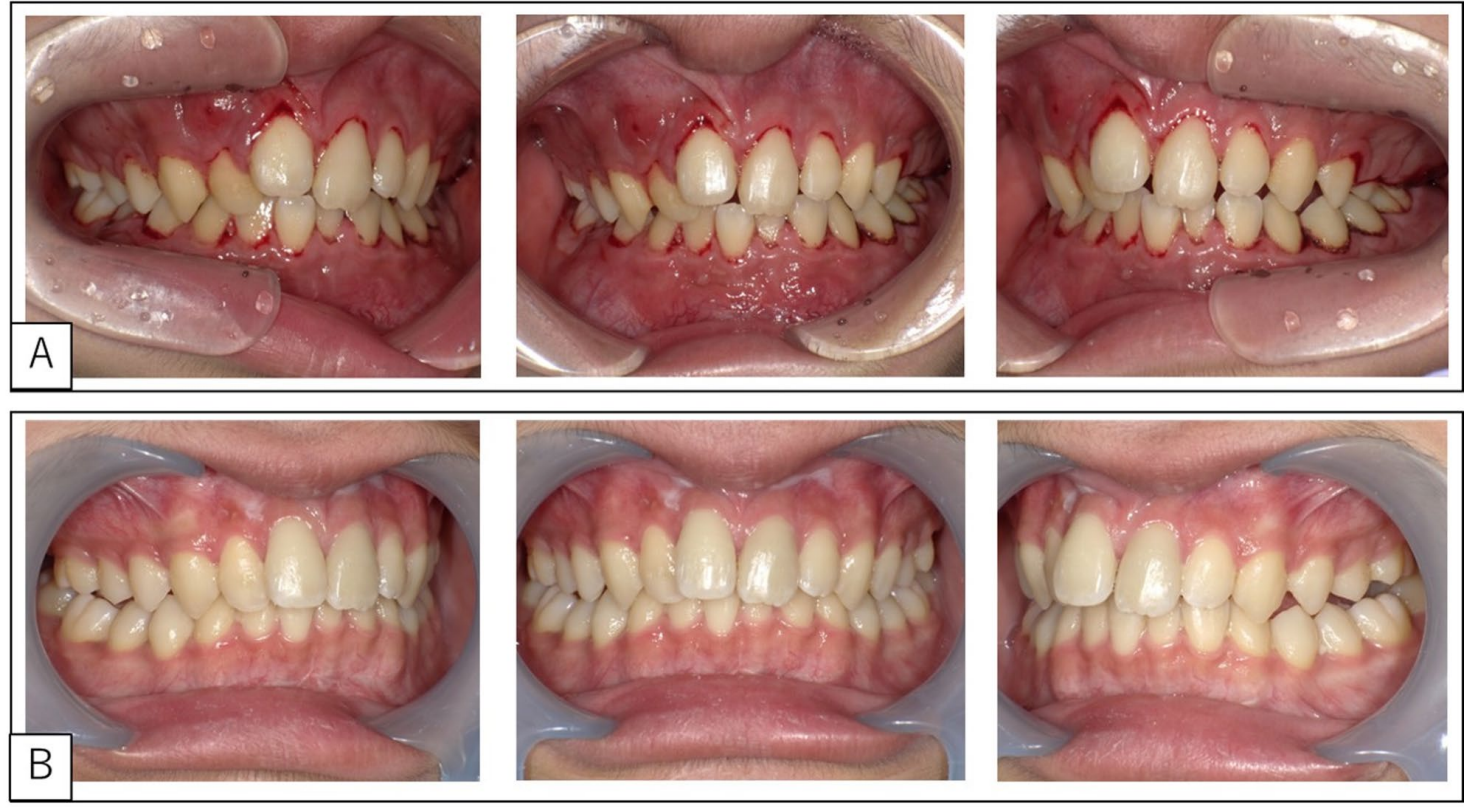
Intraoral Photograph. **A** Before treatment. Bleeding, redness, and swelling of the maxillary and maxillary gingiva were observed. **B** 116 days after the initiation of chemotherapy. All tooth movement, gingival hemorrhage, and gingival swelling resolved or improved, with no abnormalities in occlusion

His face was symmetrical. His maxillary and mandibular gingiva were bleeding, erythematous, and swollen (Fig. 2A), with significant tooth movement observed in both jaws (Table 1) and uneven bilateral occlusion of the molars.

**Table 1:** Periodontal condition

A	Mobility		3	3	3	3	3	3	3	2	1	2	3	3	
	Pocket		3	5	3	3	5	3	3	2	3	4	6	9	
		17	16	15	14	13	12	11	21	22	23	24	25	26	27
		47	46	45	44	43	42	41	31	32	33	34	35	36	37
	Pocket	4	3	3	4	4	3	3	3	3	3	3	3	5	4
	Mobility	3	3	3	3	3	2	3	3	2	2	3	3	3	3
B	Mobility		0	0	0	0	0	0	0	0	0	0	0	0	
	Pocket		2	2	2	2	2	2	2	2	2	2	2	3	
		17	16	15	14	13	12	11	21	22	23	24	25	26	27
		47	46	45	44	43	42	41	31	32	33	34	35	36	37
	Pocket	3	2	2	2	2	2	2	2	2	2	2	2	2	2
	Mobility	0	0	0	0	0	0	0	0	0	0	0	0	0	0

A: Before treatment. Periodontal pockets ranged from 3 to 9 mm. Substantial tooth movement was observed in both jaws

B: 116 days after the initiation of chemotherapy. Periodontal pockets were < 3 mm in both jaws

### Differential diagnosis, investigations, and treatment

**Fig. 3:**
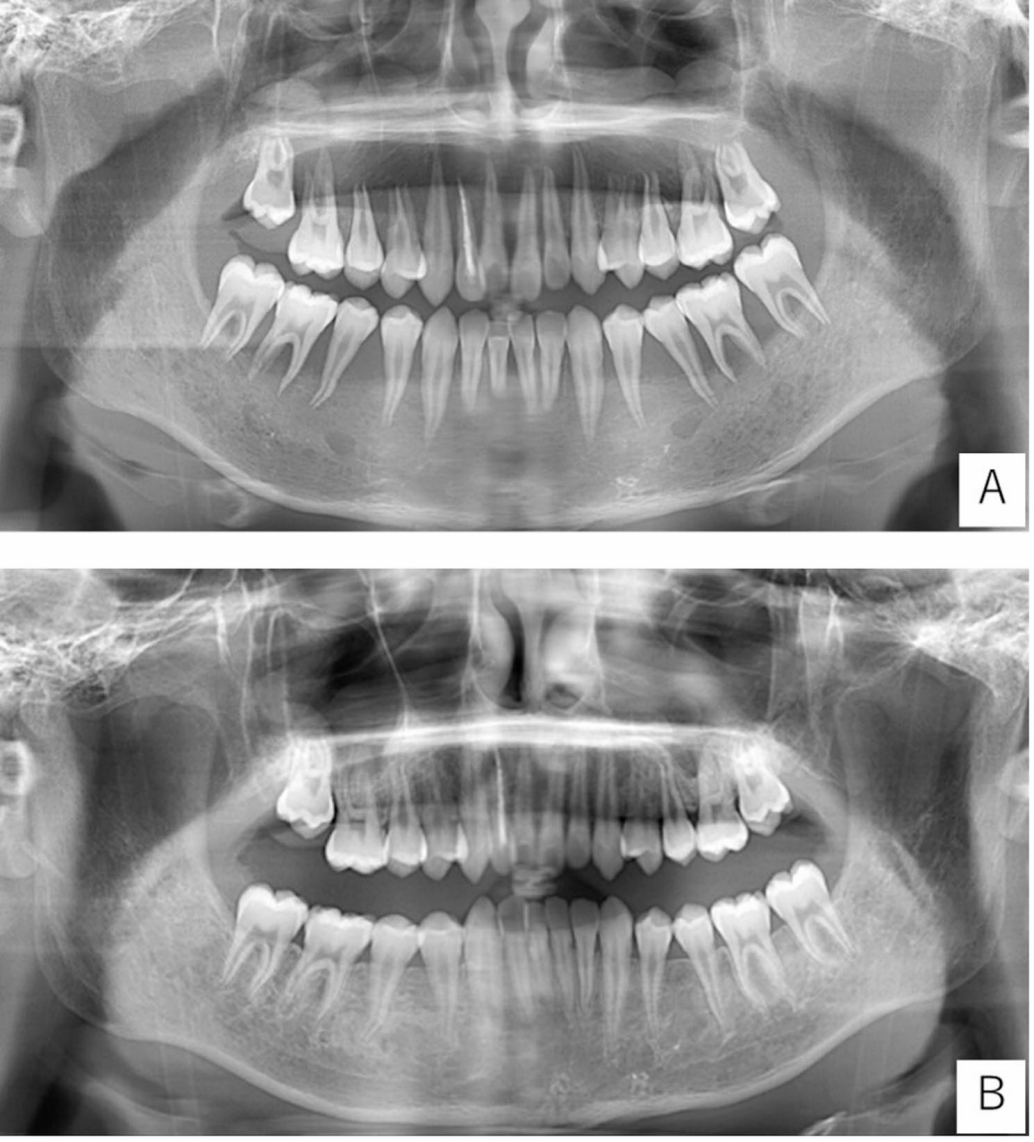
Panoramic radiographs. **A** Before treatment. **B** 116 days after the initiation of chemotherapy

**Fig. 4:**
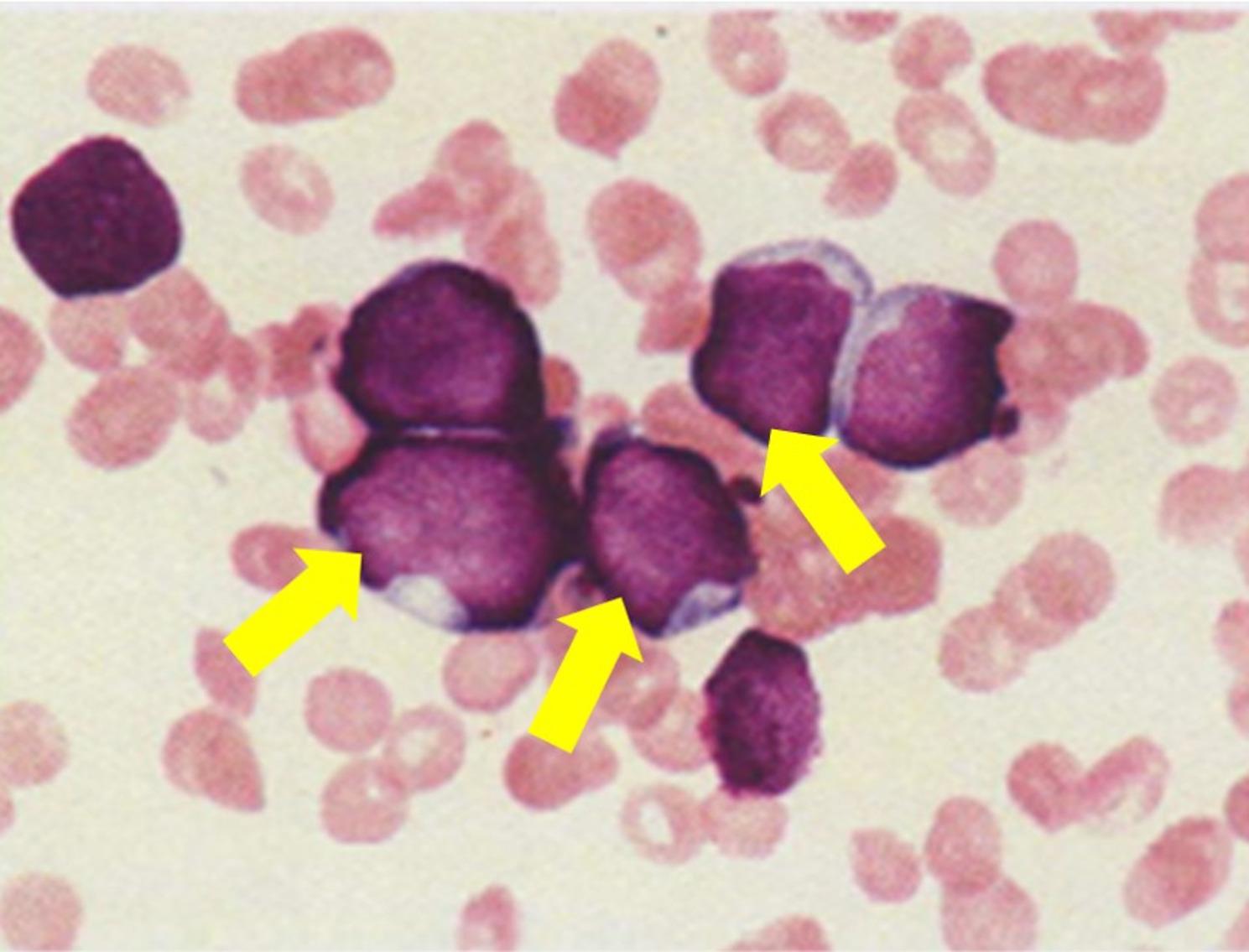
Bone marrow test results. Bone marrow smear findings: Lymphoblast with medium to large vacuoles with a high nucleus/cytoplasm ratio of 75.8% (yellow arrow)

Panoramic radiograph imaging findings on presentation showed significant alveolar bone resorption in both jaws, while the alveolar hard line, mandibular canal wall, and trabecular structure were indistinct (Fig. 3A). The patient had a white blood cell count of 15,900/µL, while other blood cell counts were decreased. In addition, blasts were observed in the peripheral blood, and C-reactive protein, lactate dehydrogenase, and ferritin levels were elevated, suggesting leukemia. Based on the patient’s systemic symptoms, including persistent low-grade fever, fatigue, and abnormal laboratory findings, blood examinations were performed by the pediatric department. ALL was diagnosed by the pediatric hematology team following comprehensive hematological evaluation and bone marrow biopsy. The dental findings, including severe gingival swelling, tooth mobility, and marked alveolar bone resorption, were therefore interpreted as manifestations of systemic leukemia rather than primary periodontal disease. A bone marrow biopsy (BMB) was then performed, which confirmed the diagnosis of B-ALL, with 75.8% of the lymphoblasts being medium to large in size and showing a high nucleus: cytoplasm ratio with vacuolation. We attributed the oral lesions to alveolar bone resorption due to B-ALL cell infiltration (Fig. 4).

### Treatment and course

On admission, IA4 remission induction therapy (vincristine 1.5 mg/m^2^ intravenous injection: days 1, 8, 15, 22, and 28; daunorubicin 30 mg/m^2^ intravenous infusion: days 1, 8, 15, and 22; L-asparaginase 5000 U/m^2^ intravenous infusion: days 5, 8, 11, 14, 17, 20, 23, and 26; methotrexate 12 mg bone marrow injection: days 5 and 26; cytarabine 30 mg bone marrow injection: days 5 and 26) was administered according to the ALL-B12 protocol. The patient was not cleaning his mouth for fear of tooth movement and general malaise, and his oral hygiene thus worsened. Therefore, oral care was continuously provided during chemotherapy. This oral management consisted of regular oral examinations, professional mechanical plaque removal (PMPR), supragingival and gentle subgingival scaling adjusted according to the patient’s hematological status, and continuous oral hygiene instruction. No local drug delivery systems or systemic antibiotics were administered specifically for periodontal treatment. Another BMB on day 14 of IA4 treatment showed that the lymphoblasts had disappeared. The patient’s gingiva had improved, with swelling and redness disappearing. The patient entered remission on day 43 of the induction therapy. On day 54, he was started on phase IB early intensification therapy (cyclophosphamide 1000 mg/m^2^ intravenous infusion: days 1 and 29; cytarabine 75 mg/m^2^ intravenous injection: days 3–6, 10–13, 17–20, and 24–27; methotrexate 12 mg bone marrow injection: days 10 and 24; cytarabine 30 mg bone marrow injection: days 10 and 24; mercaptopurine 60 mg/m^2^ oral: days 1–28) to prevent relapse. During this period, mucositis was observed in the oral cavity, probably owing to chemotherapy, but no tooth movement was observed. Fifty-three days after beginning early intensive therapy, IB, BMB showed the patient was still in remission, and intensive therapy was started to prevent relapse. An oral examination and imaging were performed 9 d after commencing intensified therapy and 116 d after beginning treatment.

### Outcome and follow-up

All tooth movement, gingival hemorrhage, and gingival swelling resolved or improved, with no abnormalities in occlusion (Fig. 2B). Periodontal pockets were < 3 mm in both jaws (Table 1). Panoramic radiographs showed bone regrowth in areas of significant alveolar bone resorption. Previously unclear structures, such as the alveolar hard line, mandibular canal wall, and bone beam structure, became well defined. On day 116 after beginning treatment, in the left mandibular molar area, which had been significantly resorbed, the jawbone had regenerated up to the tooth cervix. At initial examination, only up to approximately one-third of the root apex had been visible (Fig. 3B).

## Discussion and conclusions

Children are more likely to experience leukemias such as ALL and acute myeloid leukemia than adults [11, 12]. Fever, hemorrhage, and anemia are the three major initial signs of acute leukemia. Other findings include enlarged lymph nodes, skin symptoms, hepatosplenomegaly, and neurological symptoms, which vary according to the type of disease.

Takagi et al. [13] and Marukawa et al. [2] reported that approximately 20–35% of patients with leukemia have oral symptoms initially. Therefore, it is important to accurately understand oral symptoms of leukemia and include them in differential diagnoses of oral diseases [2, 13]. Common symptoms include gingival hemorrhage, gingival swelling, hypersensitivity, jawbone abnormalities, and tooth movement [2–8, 13] pointing to the importance of oral lesions in ALL diagnosis [14].

The patient had previously been seen by a dentist because of marked swelling and pain of the gingiva, tooth movement, and alveolar bone resorption. However, when he returned four weeks later, the inflammatory lesions, which were thought to be periodontitis, had spread further over the entire jaw and worsened; thus, the patient was referred to us. At the first presentation to our department, the patient had mild fever, malaise, and no improvement in periodontal tissue inflammation.

Confirmation of bone marrow blasts can effectively diagnose leukemia [15]. However, Okamoto et al. [6] reported a case without typical oral symptoms and in poor general condition; therefore, the diagnosis of periodontal disease, including the possibility of leukemia, should be made carefully.

Oral symptoms become more severe and oral intake becomes difficult as leukemia progresses, further affecting nutritional status and overall health. Therefore, providing adequate oral care during the treatment of leukemia is crucial to prevent secondary oral infections and health deterioration. The marked improvement in gingival condition and alveolar bone regeneration observed after chemotherapy may be attributed to several mechanisms. Resolution of leukemic cell infiltration and normalization of hematopoiesis likely reduced systemic and local inflammation, thereby restoring periodontal tissue healing capacity. In addition, pediatric patients possess a high bone remodeling potential, which may have contributed to the substantial recovery of alveolar bone once the underlying systemic disease was controlled.

To identify the cause of ALL-associated jawbone abnormalities, Takada et al. [16] performed site-specific autopsies of the mandible in 21 patients with leukemia and evaluated leukemia cell infiltration, which was confirmed in the mandibular bone marrow and gingiva (*n* = 21), alveolar bone marrow (*n* = 19), periodontal ligament (*n* = 19), and dental pulp (*n* = 20). Leukemia cells infiltrate the mandible, starting from the jaw bone marrow, then invade the alveolar bone, which supports the tooth, before subsequently invading and proliferating within the periodontal ligament and pulp.

However, in the present case, the clinical symptoms showed a clear systemic abnormality rather than localized oral disease, so a gingival biopsy was not performed to avoid the risk of spreading the disease [17]. Additionally, the panoramic radiographic examination showed a high degree of jaw bone resorption; thus, we considered it likely that the tooth movement and alveolar bone resorption were caused by leukemia cell infiltration.

In children with ALL, bone mineral loss is sometimes observed from the time of diagnosis, which is due to a decrease in bone formation markers, such as type I procollagen, C-terminal propeptide, and bone alkaline phosphatase, as well as increased bone resorption driven by parathyroid hormone-related peptide secreted by the leukemia cells. Other factors include reduced calcitrol levels, hypercalciuria, and the destruction of the sea-level chamber (metaphyseal trabecular bone) by leukemic cell infiltration [18, 19].

Many reports have detailed bone destruction due to leukemia cell infiltration, even in organs other than the jawbone; when leukemia cell infiltration is cured by remission therapy, the destroyed bones recover in correlation with the remission [9, 20].

The patient also complained of shoulder joint symptoms at initial examination and was diagnosed with a shoulder joint fracture after a visit to an orthopedic surgeon. The shoulder joint fracture was also considered to have recovered because the infiltrating leukemia cells disappeared along with the remission of ALL.

Jawbone resorbed by chronic marginal periodontitis generally does not recover; [21] however, the jaw bone will often recover in cases of leukemia-associated jaw bone resorption, as has been reported for the hands and feet [22, 23]. Even with severe bone resorption, as in the present case, recovery is likely to occur with remission. During chemotherapy, dental management in pediatric leukemia patients focuses on infection control, prevention of oral complications, and maintenance of oral function through close collaboration with the medical team. Previous reports have emphasized the importance of non-surgical periodontal therapy and careful oral hygiene management during chemotherapy. Our findings are consistent with these reports and suggest that appropriate oral care during chemotherapy may contribute to infection prevention as well as favorable periodontal and alveolar bone outcomes.

Although few reports have detailed extraction procedures performed on patients with ALL and tooth movement immediately after leukemia, the alveolar bone can completely recover, as seen here.

In Japan, many case reports mention patients with oral symptoms caused by leukemia, especially jaw bone resorption resulting in tooth movement; however, this is the first report in which a patient with highly resorbed jaw bone and tooth movement recovered with successful chemotherapy.

Future studies could systematically investigate the mechanisms and predictors of alveolar bone regeneration following chemotherapy in pediatric ALL patients. Using larger cohorts and longitudinal imaging and biomarker analyses can also yield better clinical recommendations.

In brief, we report a pediatric case of ALL including marked tooth movement and jaw bone resorption. X-ray imaging showed increased alveolar bone opacities following remission. After treatment, the tooth movement issue was resolved and occlusal function was restored.

## Data Availability

All data generated or analyzed during this study are included in this published article. No additional data are available.

## Abbreviations

ALL: Acute lymphoblastic leukemia
BMB: Bone marrow biopsy
B-ALL: B-cell acute lymphoblastic leukemia
